# Estrogen-Related Receptor Potential Target Genes in Silkworm (*Bombyx mori*): Insights into Metabolic Regulation

**DOI:** 10.3390/insects16050469

**Published:** 2025-04-29

**Authors:** Luyu Hou, Jinxin Wu, Die Liu, Haoran Xu, Hongbo Yao, Yiwen Liang, Qingyou Xia, Ping Lin, Guanwang Shen

**Affiliations:** 1 Biological Science Research Center, Southwest University, Chongqing 400715, China; hly123321@email.swu.edu.cn (L.H.); bhwjx1993@swu.edu.cn (J.W.); liudie@email.swu.edu.cn (D.L.); xhr20010430@email.swu.edu.cn (H.X.); yao20210528@email.swu.edu.cn (H.Y.); xiaqy@swu.edu.cn (Q.X.); linpingswu@swu.edu.cn (P.L.); 2 College of Sericulture, Textile and Biomass Sciences, Southwest University, Chongqing 400715, China; mu2580@email.swu.edu.cn

**Keywords:** estrogen-related receptor, estrogen-related receptor response element, metabolic regulation, physiological function, silkworm

## Abstract

Estrogen-related receptors (ERRs) are important transcription factors within the nuclear receptor family that regulate cellular energy storage and consumption by binding to estrogen-related receptor response elements (ERREs) on gene promoters. While ERRs’ role in vertebrates is well-studied, their molecular mechanisms in insect metabolism and development remain unclear. This study systematically summarizes the functions of ERRs in insects, focusing on silkworms by analyzing gene functions and comparing databases. ERRE-like elements were identified in the 2000 bp upstream promoter regions of 69 metabolism-related silkworm genes. Furthermore, electrophoretic mobility shift assays (EMSAs) revealed that ERREs within the promoters of 15 genes related to sugar, fat, and protein metabolism specifically bind to ERR. Notably, an ERRE in the promoter of the trehalose transporter 1 gene (*BmTret1*), crucial for trehalose homeostasis in insect hemolymph, exhibited significantly enhanced activity in ERR-overexpressing cells. These findings suggest that ERR is a potential regulatory factor in silkworm metabolism and refine its metabolic regulatory network. This study highlights the broader and more critical role of ERR in insects than that previously recognized, contributing to a deeper understanding of insect metabolism and its potential applications in related fields.

## Simple Summary

This study, through a combination of bioinformatics analysis and EMSA (Electrophoretic Mobility Shift Assay), confirmed that the promoter regions of 15 metabolism-related genes in the silkworm (*Bombyx mori*) can be directly regulated by Estrogen-related receptors (ERR), highlighting the potential regulatory role of ERR in key metabolic pathways of the silkworm. These findings offer new insights into the metabolic regulatory mechanisms of insects.

## 1. Introduction

Energy metabolism involves the conversion of nutrients into cellular energy, regulated by the synergistic action of multiple transcription factors and co-regulatory factors [1]. Estrogen-related receptors (ERRs) are key transcription factors within the nuclear receptor family, notable for their lack of natural ligands [2]. ERRs regulate target gene expression by binding to ERR elements (ERREs) located in the promoter regions of these genes through their DNA-binding domains (DBDs) [3]. In vertebrates, ERRs function by binding to ERREs either as dimers or monomers to drive gene activation [4]. Additionally, it has been demonstrated that different ERR subtypes can form heterodimers [5]. Current studies reveal that genomic data from insects such as *Drosophila melanogaster*, *Bombyx mori*, *Teleogryllus emma* and *Apis cerana cerana* consistently indicate the presence of only a single ERR subtype, with no evidence of subtype diversification [6,7,8,9]. Consequently, no additional ERR isoforms exist to serve as heterodimer partners for this specific ERR subtype. Currently, the consensus sequence of the most common motif in ERR generally corresponds to “TCAAGGTCA” and is relatively conserved across different species [3]. Structural comparative analysis revealed that while both ERREs and mammalian estrogen response elements (EREs) contain the signature recognition motif “AGGTCA”, ERREs lack the palindromic structural features characteristic of EREs, being equivalent to half of the palindromic configuration. Following the completion of the *Drosophila melanogaster* genome sequencing, which confirmed the absence of estrogen receptor (ER) in insects and identified only ERR, no ER homologs have been detected in the *Bombyx mori* genomic databases [10,11]. The target genes of ERR are involved in various biological processes, with a primary focus on mediating intracellular energy metabolite signaling and regulating cellular energy metabolism [12].

In vertebrates, ERRs exist in three isoforms—ERRα, ERRβ, and ERRγ [13]—playing key roles in energy metabolism and mitochondrial biogenesis. ERRα activates the expression of fatty acid oxidation-associated genes, such as medium-chain acyl-CoA dehydrogenase (MCAD) and carnitine palmitoyltransferase 1 (CPT1), promoting the β-oxidation process [14]. In cancer cells, ERR overexpression enhances anaerobic respiration, leading to lactate accumulation, while ERR knockdown reduces glucose uptake and inhibits aerobic glycolysis and cell growth [15]. ERRα overexpression also promotes glycolytic metabolism and regulates pyroptosis via the NOD-like receptor family pyrin domain containing 3/cysteinyl aspartate specific proteinase-1/gasdermin D (NLRP3/caspase-1/GSDMD) pathway in endometrial cancer cells [16]. Knockdown of ERRβ interferes with glucocorticoid receptor (GR)-induced gene expression, contributing to glucocorticoid (GC) resistance in vitro and in vivo [17]. Additionally, ERRγ dysregulation affects glucose metabolism, with ERRγ-expressing mice exhibiting glucose intolerance and impaired insulin secretion [18]. ERRγ also regulates lysyl oxidase (LOX) expression in hepatocytes. LOX, a copper-dependent extracellular enzyme, is crucial for protein cross-linking in the extracellular matrix and plays a role in metabolic processes such as liver regeneration and fibrosis [19]; Furthermore, studies have identified 705 functional mitochondrial proteins in vertebrate mitochondria, including ATP-related genes regulated by ERRs. They include mitochondrial fusion protein 2 (*Mfn2*), nuclear respiratory factor 1/2 (*NRF1/2*), and ATP synthase β subunit (*ATP5B*), which encode proteins involved in mitochondrial biogenesis and function [20].

**Figure 1:**
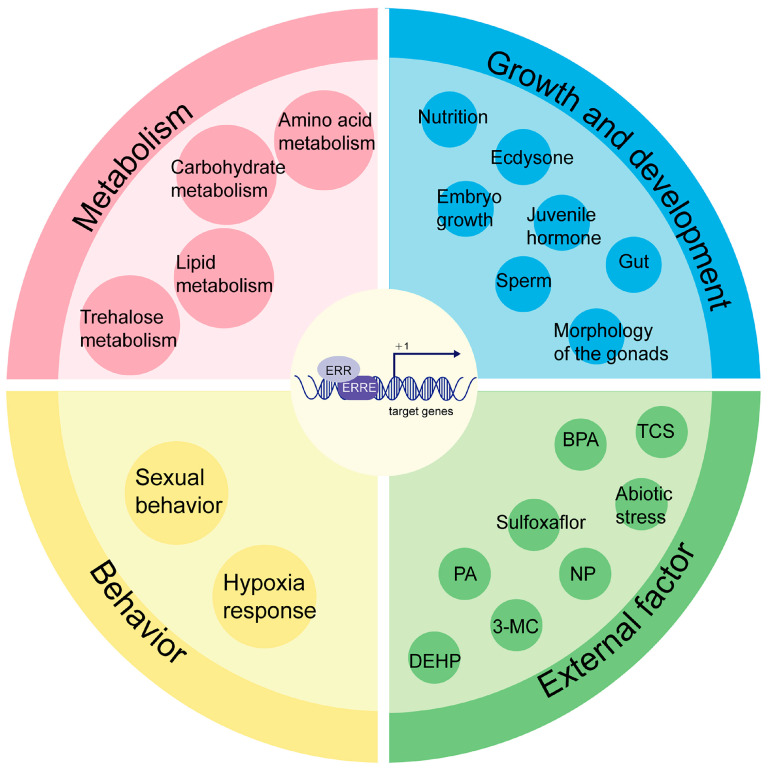
Summary of ERR functions in insects. TCS: triclosan; BPA: bisphenol A; PA: 4-nonylphenol; NP: nonylphenol; 3-MC: 3-Methylcatechol; DEHP: di(2-ethylhexyl)phthalate.

The glycolytic process in insects such as *Drosophila melanogaster*, *Bombyx mori*, and aphids is also modulated by ERR. In *Drosophila melanogaster*, the loss of ERR results in decreased ATP levels, downregulation of glycolytic pathway genes, increased trehalose and glycogen levels, reduced triglyceride (TAG) levels, and diminished lipid content in adult fat bodies. These findings highlight the regulation of both sugar and lipid metabolism by ERR in *Drosophila melanogaster* [21,22]; Additionally, *Drosophila melanogaster* ERR interacts with hypoxia-inducible factors (HIFs), key regulators role of the cellular hypoxic response, to modulate the transcriptional activity of metabolism-related target genes [23]. In *Bombyx mori* embryos, glycolysis-related genes such as hexokinase (*HK*), pyruvate kinase (*PK*), and phosphofructokinase (*PFK*) are regulated by ERR. Suppressing ERR expression through double-stranded RNA interference delays the hatching time of silkworm embryos [24], whereas upregulating ERR accelerates metabolism, enhancing embryo mobility after hatching [25]. ERR also plays a significant role in the growth, development, and reproduction of insects. In *Drosophila melanogaster,* reduced ERR expression in seminal vesicles leads to abnormal sperm development and a significant decline in sperm count [26]; Similarly, ERR deficiency in aphids reduces female offspring production [27], while male *Agrotis ipsilon* with ERR deficiencies exhibit abnormal courtship behaviors [28]; Furthermore, ERR regulates vitellogenin gene expression in silkworms [6] and influences the expression of juvenile hormone/20-hydroxyecdysone (JH/20E) hormone signaling-related genes in *Nilaparvata lugens*, affecting molting and ovarian development [29]. In summary, the function of ERRs in insects identified to date primarily focus on several aspects, including energy metabolism, growth and development, behavioral regulation, and stress responses (Table 1, Figure 1). ERR has been shown to regulate lipid and carbohydrate metabolism in insects such as *Bombyx mori*, *Drosophila*, *Myzus persicae*, and *Aedes aegypti*. Additionally, ERR can modulate insect hormones, such as the 20-hydroxyecdysone, juvenile hormone, and insulin signaling pathways, as observed in species such as *Nilaparvata lugens* and *Polyrhachis vicina Roger*. These interactions influence critical processes such as growth, development, and reproduction. Furthermore, ERR has been implicated in behavioral regulation, as evidenced in species like *Agrotis ipsilon* and *Drosophila melanogaster*, and in stress responses observed in *Chironomus riparius* and *Apis cerana cerana*.

Although insects possess only one ERR, its functional significance is comparable to that in vertebrates and appears even more widespread and complex. Despite advances in understanding ERR functions in insects, comprehensive molecular studies on the underlying mechanisms remain limited. This study systematically summarizes the roles of ERR in insects, using domesticated silkworms as a model organism. By scanning and identifying ERREs in the promoters of metabolically related genes in silkworms, we analyzed the molecular mechanisms of ERR functions and extended the metabolic regulatory network of ERR in insects.

## 2. Materials and Methods

### 2.1. Insects

The silkworm variety D9L was obtained from our research group and reared on fresh mulberry leaves at 25 ± 1 °C under a 12-h light/12-h dark photoperiod. The suitable humidity range for silkworm rearing is 85–90% during the first instar, decreasing by 5% with each subsequent instar, and reaches approximately 70–75% by the fifth instar.

The overexpression ERR silkworm prepared by our research group contains the recombinant plasmid piggybac (3×p3-dsRed-SV40, Hr3-A4-BmERR-SV40). The dsRed positive expression of G1 embryos and moths was screened under fluorescence microscope (Olympus, Kyoto, Japan). The transgenic lines were screened and named OE-BmERR for subsequent detection experiments.

### 2.2. Sample Collection and Preparation

#### 2.2.1. Extraction of Tissue Protein

Tissue samples (100 mg) from the head, hemolymph, midgut, epidermis, and fat body were ground into a fine powder using liquid nitrogen in a Freezing Mill 6875D (PYNN, Boston, MA, USA). The powdered tissue was transferred to a 1.5 mL centrifuge tube and 500 μL of RIPA lysis buffer (Beyotime Biotechnology, Shanghai, China) supplemented with 1 mM protease inhibitor was added. The mixture was incubated on ice for 30 min. After incubation, the samples were centrifuged at 16,100× *g* for 15 min at 4 °C. The supernatant, containing the extracted protein solution, was collected and stored. Protein concentrations were determined using a BCA protein assay kit (Beyotime Biotechnology, Shanghai, China).

#### 2.2.2. Extraction of Nuclear Protein

Nuclear proteins were extracted from the silkworms using the Nucleoprotein Extraction Kit (Beyotime Biotechnology, Shanghai, China) according to the manufacturer’s instructions. Briefly, the midguts of fifth-instar silkworms on day 3 were ground into powder using liquid nitrogen in a pre-chilled mortar and transferred to a 1.5 mL centrifuge tube. A homogenate was prepared by mixing cytoplasmic protein extraction reagents A and B at a 20:1 ratio, supplemented with PMSF at a final concentration of 1 mM. For every 60 mg of tissue, 200 µL of the homogenate was added, and the mixture was homogenized thoroughly on ice. The homogenate was transferred to a plastic centrifuge tube and placed in an ice bath for 15 min and then centrifuged at 1500× *g* for 5 min at 4 °C. The supernatant was discarded, and the sediment was washed to remove residual supernatant. Nuclear protein extraction reagent containing PMSF (50 μL) was added to the sediment, and the mixture was vortexed for 15–30 s. This step was repeated every 1–2 min over 30 min while keeping the sample on ice. Finally, the mixture was centrifuged at 12,000–16,000× *g* for 10 min at 4 °C, and the supernatant containing nuclear proteins was transferred to a pre-chilled tube.

### 2.3. Western Blot

Protein samples (20 μg) were separated on a 12% (*w*/*v*) polyacrylamide gel using SDS-PAGE and subsequently transferred to a PVDF membrane [41]. The membrane was blocked with 5% nonfat dry milk in TBST and incubated with specific primary antibodies against BmERR (This BmERR antibody is rabbit-derived, produced and preserved by our research team) or α-tubulin (Beyotime Biotechnology, Shanghai, China). Horseradish peroxidase (HRP)-conjugated goat anti-rabbit IgG (Beyotime Biotechnology, Shanghai, China) was used as the secondary antibody for BmERR (the antibody against BmERR was prepared and stored by our research team), and goat anti-mouse IgG (Beyotime Biotechnology, Shanghai, China) was used as the secondary antibody for α-tubulin. The primary and secondary antibodies were diluted 1:10,000 in TBST containing 2% nonfat dry milk. Protein detection was performed using enhanced chemiluminescence reagents, and images were captured using a Clinx ChemiScope 3400 Mini machine.

### 2.4. Bioinformatics Analysis

Sequences of 2000 bp upstream of genes associated with sugar, lipid, amino acid, and carbohydrate metabolism were retrieved from the silkworm genome database Silk DB 3.0 (http://www.silkdb.org/silkd-b/, (accessed on 27 April 2025)) and NCBI (https://www.ncbi.nlm.nih.gov/, (accessed on 27 April 2025)). Using the JASPAR database (http://jaspar.genereg.net/, (accessed on 27 April 2025)), ERREs in promoter regions were predicted based on the motif sequences of ERR (MA2226.1) and Esrrg (MA0643.1), with a relative profile score threshold set at 80%.

### 2.5. Electrophoretic Mobility Shift Assay

To investigate the binding of ERR proteins to metabolism-related genes, probes were designed based on predicted ERRE positions within the silkworm genome (Tables S1 and S2). Probes were biotinylated at the 3′ end and annealed to form double-stranded probes. The EMSA/gel shift experiment was conducted using the ERR DBD protein expressed in *E. coli* and intestinal nuclear proteins, following the manufacturer’s protocol (Beyotime Biotechnology, Shanghai, China). After preparing the reaction mixtures, samples were incubated at 25 °C for 25 min. The samples were then loaded onto a 5% native polyacrylamide gel and subjected to electrophoresis in 1 × TBE buffer (45 mM Tris-borate, 1 mM EDTA, pH 8.3). Following electrophoresis, the protein-nucleic acid complexes were transferred onto a nylon membrane (Roche, Indianapolis, IN, USA). The membrane was incubated with enhanced chemiluminescent reagents (Thermo Fisher Scientific, Waltham, MA, USA) and visualized using a Clinx ChemiScope 3400 Mini machine.

### 2.6. Vector Construction

The promoter sequence of *BmTret1* (−2000 bp to +100 bp upstream of the gene) was amplified from the silkworm genome. This amplified promoter sequence was excised using the restriction endonucleases *XhoI* and *NheI* and ligated into the pGL3-basic vector (Promega, Madison, WI, USA). The constructed vector, named pGL3-Tret1p, includes a firefly luciferase reporter gene that reflects promoter activity through luciferase expression levels.

### 2.7. Cell Transfection and Luciferase Assay

The BmE cell line (maintained in this study) was cultured at 27 °C in Grace insect medium (Gibco, Grand Island, NE, USA) supplemented with 10% fetal bovine serum (FBS; Gibco, Grand Island, NE, USA), penicillin G (200 U/mL), and streptomycin (200 U/mL). Cells were seeded in 24-well plates and incubated for 12 h before transfection. A transfection mixture containing 1 μg of recombinant plasmid pGL3-Tret1p, 0.1 μg of internal control plasmid pRL-78 ML (containing only a TATA box and no cis-regulatory elements, stored in our laboratory), and 3 μL of Lipofectamine 2000 (Invitrogen, Carlsbad, CA, USA) was added to the wells. In addition, another set of mixtures containing 0.5 μg of pSL1180-HR3-A4-BmERR-SV40 (preserved in our laboratory), 0.5 μg of pGl3-Tret1p, 0.1 μg of pRL-78 ML, and 3 μL of Lipofectamine 2000 was also prepared for co-transfection. After 8 h, the transfection mixture was replaced with 500 μL of fresh insect medium containing 10% FBS. The cells were harvested 48 h post-transfection, and luciferase activity was measured using a dual-luciferase reporter assay kit (Yeasen Biotechnology, Shanghai, China).

### 2.8. Statistical Analysis

Results were expressed as the mean ± SD of three biological replicates. Statistical analyses were performed using Microsoft Excel 2019 (Microsoft Corporation, Washington, DC, USA) and GraphPad Prism 8.2 software (GraphPad Software, San Diego, CA, USA). The Shapiro-Wilk test confirmed that the data followed a normal distribution. Given that the analysis involved comparing the mean differences between two independent groups, Student’s *t*-test was selected as the appropriate statistical method and * p < 0.05, ** p < 0.01, *** p < 0.001, and **** p < 0.0001.

## 3. Results

### 3.1. Detection of ERR Expression and Screening of Target Genes in Silkworm

**Figure 2:**
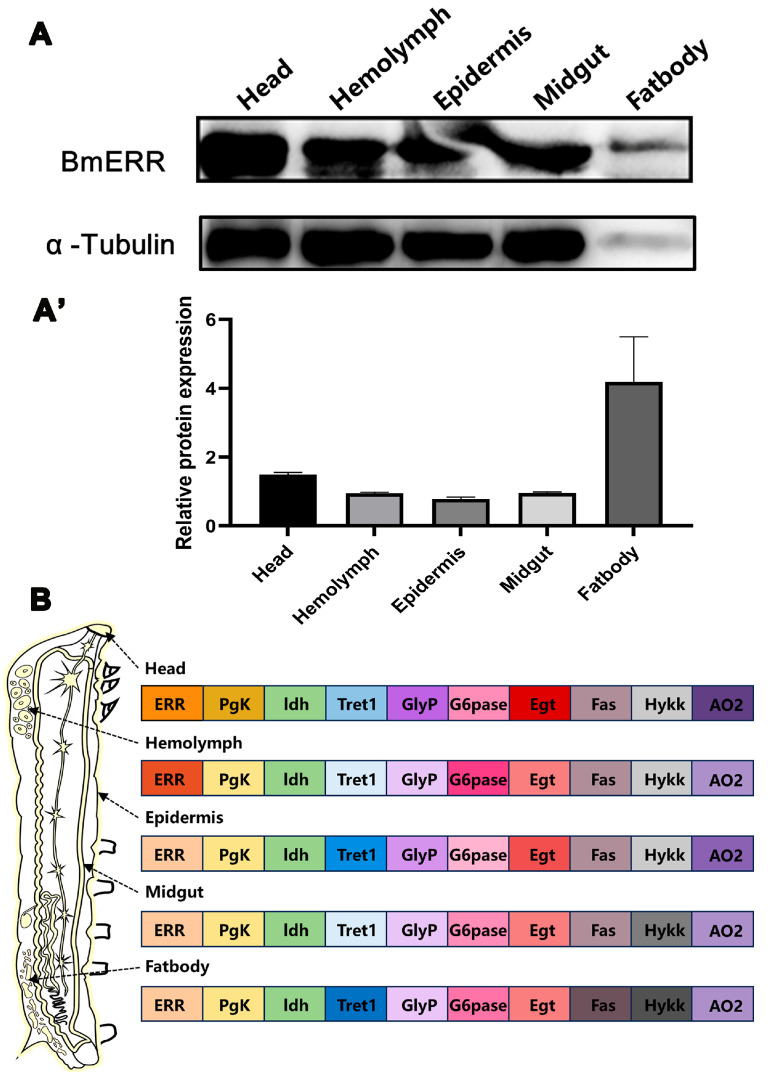
Tissue-specific expression profiles of BmERR protein and metabolism-related genes in silkworm. (**A**,**A’**) The expression of BmERR protein in the head, hemolymph, epidermis, midgut, and fat body was detected using Western blotting. (**B**) Expression profiles of *ERR*, *PgK*, *ldh*, *Tret1*, *GlyP*, *G6pase*, *Egt*, *Fas*, *Hykk*, and *AO2* genes in the primary tissues of adult silkworms (the color gradient from dark to light represents expression levels from high to low). Data referenced from Silkbase (https://silkbase.ab.a.u-tokyo.ac.jp/cgi-bin/index.cgi, (accessed on 27 April 2025)). *PgK*: Phosphoglycerate kinase; *ldh*: Isocitrate dehydrogenase; *Tret1*: Trehalose transporter1; *GlyP*: Glycogen phosphorylase; *G6pase*: Glucose-6-phosphatase; *Egt*: UDP-glycosyltransferase; *Fas*: Fatty acid synthase; *Hykk*: Hydroxylysine kinase; *AO2*: Aldehyde oxidase.

To explore the metabolic functions of BmERR, we initially profiled its expression across major metabolic tissues. On day 3 of the fifth instar, the head, blood cells, epidermis, midgut, and fat body of the silkworms exhibited BmERR expression (Figure 2A). Additionally, metabolism-related genes such as phosphoglycerate kinase (*PgK*), lactate dehydrogenase (*ldh*), *Tret1*, glycogen synthase (*GlyP*), glucose-6phosphatase (*G6pase*), UDP-glucosyltransferase (*Egt*), fatty acid synthase (*Fas*), hydroxyl lysine kinase (*Hykk*), and aconitate decarboxylase 2 (*AO2*) were expressed at varying levels in these tissues (Figure 2B, SilkBase data (https://silkbase.ab.a.u-tokyo.ac.jp/cgi-bin/index.cgi, (accessed on 27 April 2025)).

**Figure 3:**
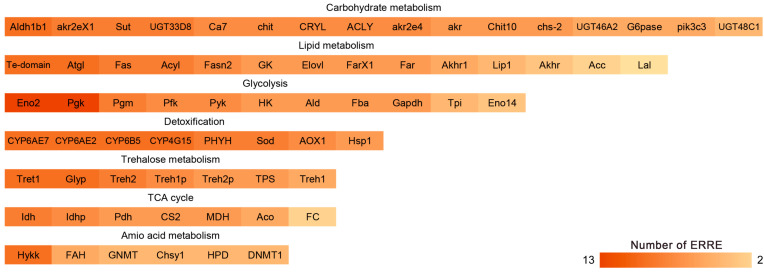
Number of ERRE-like elements with a score greater than 80 on the promoters of metabolism-related genes.

To analyze the regulatory relationship between ERR and the expression of these genes, the JASPAR database was used to predict ERRE-like elements in the promoter regions located 2000 bp upstream of 69 metabolism-related genes. These genes were associated with lipid metabolism, glycolysis, carbohydrate metabolism, detoxification metabolism, trehalose metabolism, the tricarboxylic acid cycle, and amino acid metabolism. Promoter sequences of the 69 genes contained varying numbers of ERRE-like elements when the correlation coefficient exceeded 80% (Figure 3, Table S1).

**Figure 4:**
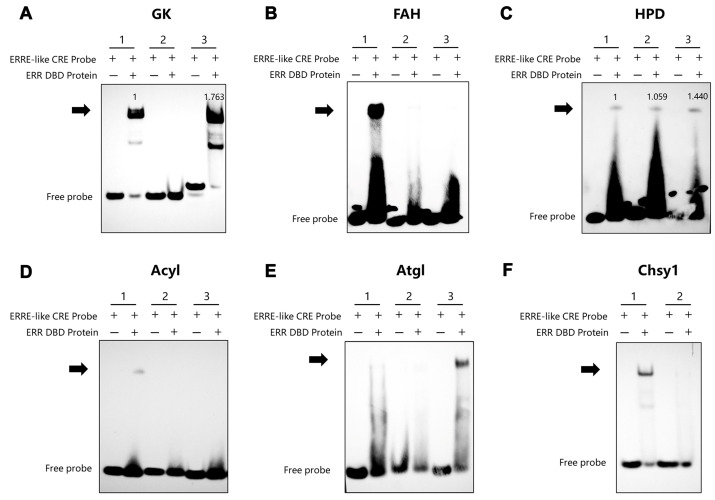
Verification of BmERR DBD protein binding to promoters of lipid and amino acid metabolism-related genes. (**A**–**F**) Electrophoretic mobility shift assays using the BmERR DBD protein and ERR CREs related to lipid and amino acid metabolism genes. ERRE-like CRE Probe: Estrogen-related receptor response element core element; *GK*: Glycerol kinase; *Acyl*: Acyl-CoA; *Atgl*: Adipose triglyceride lipase; *FAH*: Fumarate hydratase; *HPD*: 4-Hydroxyphenylpyruvate dioxygenase; *Chsy1*: Chondroitin sulfate synthase 1. 1, 2, 3: ERRE-like CRE1 Probe, ERRE-like CRE2 Probe, ERRE-like CRE3 Probe. The numerical values on the blot represent normalized grayscale values referenced to the first binding-positive lane.

Based on the metabolic classification of the genes, we selected key genes from the following pathways for validation: lipid metabolism, trehalose metabolism, amino acid metabolism, and carbohydrate metabolism. For experimental validation, two to four ERRE components exhibiting relatively high rating coefficients were systematically selected. Biotin-labeled ERRE-like elements from the promoters of key enzymes involved in these pathways were incubated with recombinant BmERR DBD protein (Table S2). EMSA results revealed that there were significant binding phenomena in 15 genes (Table 2). Among them, glycerol kinase (*GK*) (Figure 4A), acyl-CoA (*Acyl*) (Figure 4D), and adipose triglyceride lipase (*Atgl*) in lipid metabolism (Figure 4E), as well as fumarylacetoacetate hydrolase (*FAH*) (Figure 4B), 4-hydroxyphenylpyruvate dioxygenase (*HPD*) (Figure 4C), and chondroitin sulfate synthase 1 (*Chsy1*) (Figure 4F) in amino acid metabolism all had obvious significant supershifted bands.

**Figure 5:**
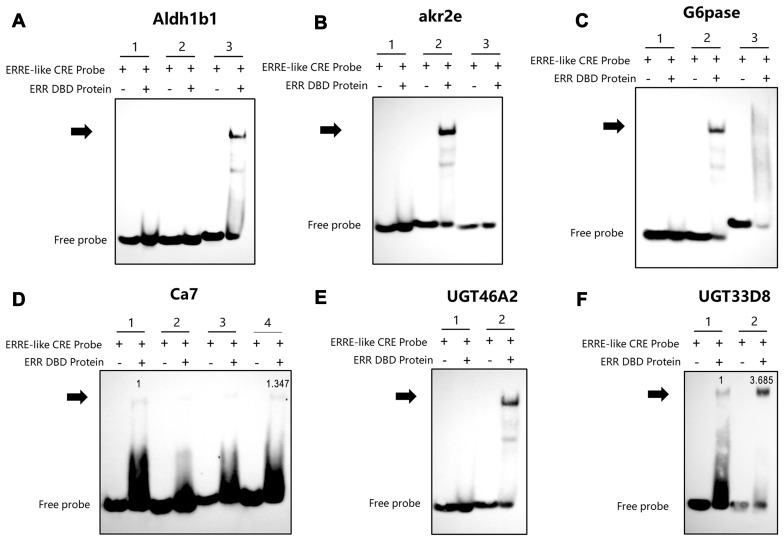
Verification of BmERR DBD protein binding to promoters of carbohydrate metabolism-related genes (**A**–**F**) Electrophoretic mobility shift assays using the BmERR DBD protein and ERR CREs related to carbohydrate metabolism genes. ERRE-like CRE Probe: estrogen-related receptor response element core element; *Aldh1b1*: aldehyde dehydrogenase 1 B1; *Akr2e*: Aldo-keto reductase AKR2E4-like; *G6pase*: glucose-6-phosphatase; *Ca7*: carbonic anhydrase 7; *UGT46A2*: UDP-glucosyltransferase; *UGT33D8*: UDP-glucosyltransferase. 1, 2, 3, 4: ERRE-like CRE1 Probe, ERRE-like CRE2 Probe, ERRE-like CRE3 Probe, ERRE-like CRE4 Probe. The numerical values on the blot represent normalized grayscale values referenced to the first binding-positive lane.

For carbohydrate metabolism, aldehyde dehydrogenase 1 B1 (*Aldh1b1*) (Figure 5A), aldo-keto reductase family 2 member E4 (*Akr2e*) (Figure 5B), glucose-6-phosphatase (*G6pase*) (Figure 5C), carbonic anhydrase 7 (*Ca7*) (Figure 5D), UDP-glucuronosyltransferase *UGT46A2* (Figure 5E), and *UGT33D8* precursor (Figure 5F) all exhibited significant lagging bands, indicating that ERR may regulate the promoter activity of these genes.

**Figure 6:**
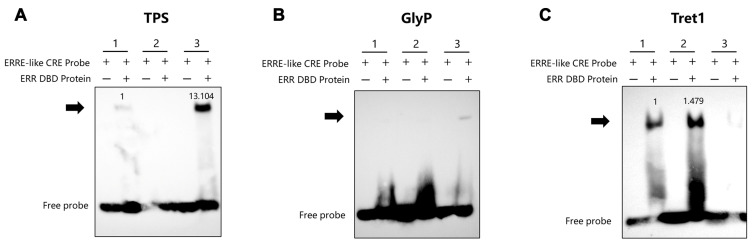
Verification of BmERR DBD protein binding to promoters of trehalose metabolism-related genes. (**A**–**C**) Electrophoretic mobility shift assays using the BmERR DBD protein and ERR CREs related to trehalose metabolism. Competitive probes were added to confirm specific binding to the BmERR DBD protein. ERRE-like CRE Probe: estrogen-related receptor response element core component; *TPS*: trehalose-6-phosphate synthase; *GlyP*: glycogen phosphorylase; *Tret1*: trehalose transporter 1. 1, 2, 3: ERRE-like CRE1 Probe, ERRE-like CRE2 Probe, ERRE-like CRE3 Probe. The numerical values on the blot represent normalized grayscale values referenced to the first binding-positive lane.

Furthermore, the promoters of trehalose-6-phosphate synthase (*TPS*) (Figure 6A), glycogen phosphorylase (*GlyP*) (Figure 6B) and trehalose transporter 1 (*Tret1*) (Figure 6C) also contained elements interacting with ERR. These findings indicate that BmERR plays a regulatory role in silkworm trehalose metabolism.

### 3.2. Identification of BmERR Target Genes

**Figure 7:**
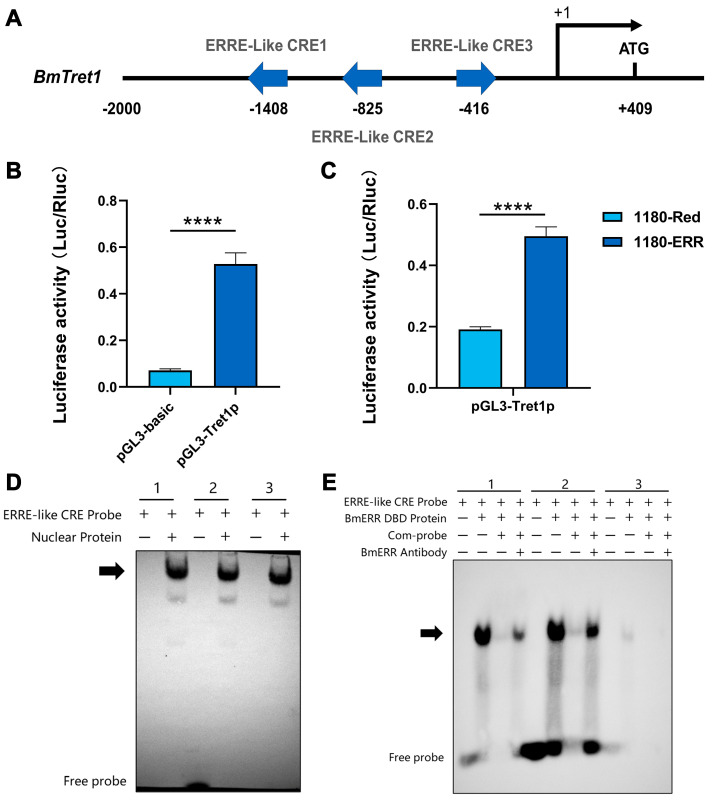
BmERR specifically binds to the promoter of *BmTret1*. (**A**) Analysis of upstream promoters of *BmTret1* and ERR CREs+1” represents the transcription start site (TSS), while “−1408, −825, −416” indicate the distances of the ERR CREs from the transcription start site. (**B**) Relative luciferase activity of the *BmTret1* promoter in BmE cells (Two-tailed Student’s *t* test: **** p < 0.0001; at least three biological replicates). (**C**) BmERR enhances luciferase activity driven by the BmTret1 promoter in BmE cells (Two-tailed Student’s *t* test: **** p < 0.0001; at least three biological replicates). (**D**) Electrophoretic mobility shift assay of nuclear proteins extracted from the midgut tissue of D9L, five days old silkworms, with three ERR CREs. (**E**) The specific binding of the BmERR to three ERR CREs was validated by electrophoretic mobility shift assay. Error bars are shown as mean ± SEM (*n* = 3). Significant differences are indicated by **** p < 0.0001. 1, 2, 3: ERRE-like CRE1 Probe, ERRE-like CRE2 Probe, ERRE-like CRE3 Probe.

Unlike vertebrates, insects possess a unique trehalose metabolism. Within this system, trehalose transporters play a crucial role by transporting trehalose, ensuring energy is promptly delivered to energy-intensive tissues, and regulating the insect’s trehalose balance, all of which hold substantial research significance. So, we selected the *BmTret1* for further investigation. The promoter region located 2000 bp upstream of the *BmTret1* gene contains three ERRE-like (highest score) (Figure 7A). This promoter region demonstrated significant promoter activity (Figure 7B). To analyze the relationship between BmERR and promoter activity, BmERR was overexpressed in BmE cells, which significantly increased *BmTret1* promoter activity (Figure 7C). In silkworm organs with active trehalose metabolism, nuclear proteins bound to ERRE-like elements 1, 2, and 3 in the fatbody (Figure 7D). Incubation of biotin-labeled ERRE-like1 and ERRE-like2 with BmERR DBD protein (prepared and stored by our research group) produced noticeable lagging bands (Figure 6C). Competitive probes and BmERR antibodies significantly weakened these binding signals (Figure 7E). These findings indicate that BmERR plays a regulatory role in silkworm trehalose metabolism.

**Figure 8:**
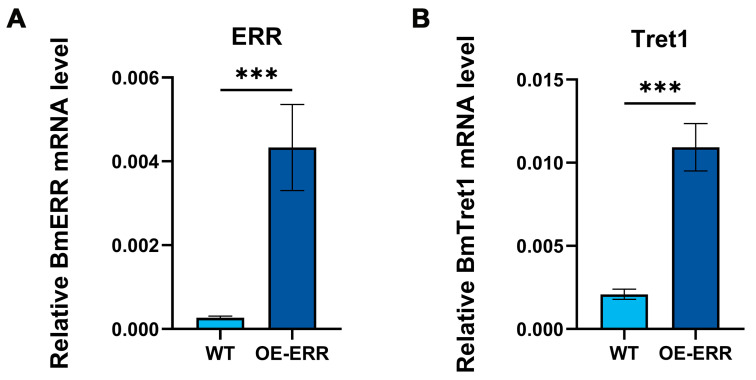
The expression of *BmTret1* is upregulated by BmERR in OE-ERR silkworm. (**A**,**B**) The qRT-PCR analysis of BmERR and *BmTret1* mRNA after overexpression of ERR in silkworm. WT: wild type; OE-ERR: Overexpression of ERR in silkworm. Error bars are shown as mean ± SEM (*n* = 3). Significant differences are indicated by *** p < 0.001.

To assess if BmERR regulates *BmTret1* transcription, we examined ERR and Tret1 expression in day-3 fifth-instar silkworms overexpressing ERR. Results showed that BmERR overexpression (Figure 8A) significantly upregulated endogenous *BmTret1* expression (Figure 8B). This synchronized expression pattern provides compelling evidence for BmERR-mediated transcriptional activation of *BmTret1*.

## 4. Discussion

The ERR is a key regulatory factor in insect growth, development, and metabolism. Its functional network spans energy metabolism, reproductive and developmental processes, morphogenesis, metamorphosis, signaling pathways, and behavioral regulation. Insects rely on energy metabolism to efficiently convert ingested nutrients into cellular energy and structural components [42]. The energy metabolism pathway of insects produces energy by decomposing sugars, carbohydrates, lipids and amino acids to support their growth, movement, development and reproduction. Fumarylacetoacetate Hydrolase Fumarylacetoacetate hydrolase (FAH) is a key enzyme in the tyrosine metabolic pathway in insects. The absence of FAH will also cause metabolic disorders and affect the normal growth and development of insects [43]; Glycerol-3-phosphate dehydrogenase (GPDH) and glycerol kinase (GK) isozymes are linked to the production of a high quantity of glycerol as a rapid cold hardening (RCH) factor, and glycerol as main cryoprotectant plays an important role in survival throughout the cold period in this quarantine pest studied [44]; Multiple mating of *Ophraella communa* can significantly increase the reproductive capacity of female insects. It was found that carbonic anhydrase showed obvious male-biased expression, and the reproductive capacity decreased after knockout of this gene [45]. RNA interference (RNAi)-mediated knockdown of trehalose synthase in *Nilaparvata lugens* significantly impairs its feeding behavior [46]. Numerous studies have shown that insect ERR can regulate various energy—metabolism pathways and functions as a master switch for energy regulation. These metabolism-related genes play crucial regulatory roles in insects. This study analyzed potential ERRE elements in promoters of metabolism-related genes. Genes were selected for EMSA validation based on predicted ERRE element scores and their positions in metabolic pathways. Results indicated that gene promoters in lipid metabolism, trehalose metabolism, amino acid metabolism, and carbohydrate metabolism pathways have fragments that bind to BmERR. However, considering EMSA limitations and gene selection restrictions, more refined experiments are needed to confirm the exact impact of ERR on these metabolic pathways. Furthermore, although we conducted preliminary probe binding assays, but whether ERR specifically binds to the response elements in these promoters to regulate the expression of these genes needs to be demonstrated through more refined experiments. For example, introducing competitive probes or mutating these elements to detect their binding difference with ERR.

Lepidopterans, which include silkworms, play a pivotal role in controlling agricultural pests. Among lepidopterans, silkworms are a preferred model organism due to their ease of laboratory rearing, short lifecycle, high reproductive output, well-defined genetic background, and low maintenance costs [47]. This study focuses on silkworms to analyze the presence of ERR-binding site-like elements in the promoters of 69 metabolism-related genes. Specifically, promoters of key enzyme genes involved in lipid, amino acid, and carbohydrate metabolic pathways in silkworms contain nucleic acid sequences capable of binding ERR. This suggests that ERR may influence the promoter activity of these genes.

Insects, unlike vertebrates, use trehalose as their primary hemolymph sugar, playing a crucial role in growth, development, molting, and metamorphosis. The transport of trehalose into and out of silkworm tissues is mediated by the Tret1, which maintains trehalose homeostasis in insects [48]. By regulating intracellular trehalose concentration, insects protect their cells from potential damage [49]. In this study, we first identified putative ERR-binding elements in the *BmTret1* promoter through bioinformatics analysis. Subsequent dual-luciferase reporter assays demonstrated direct binding between BmERR and the *BmTret1* promoter, while electrophoretic mobility shift assays further suggested that its transcriptional activity may be regulated by BmERR. Notably, functional ERREs were also detected in the promoters of two key trehalose metabolic genes, *BmGlyP* and *BmTPS*. Our previous study demonstrated that overexpression of BmERR in silkworms upregulates the expression of *BmTreh*, promotes the breakdown of trehalose into glucose, and consequently increases glucose levels in the hemolymph. However, no significant change in trehalose content was observed in the hemolymph during measurement [31]. Mechanistically, in silkworms, BmERR overexpression exhibits dual regulatory roles: promoting *BmTreh*-mediated trehalose decomposition in hemolymph to generate glucose for enhanced energy production, and potentially facilitating *BmTret1*-mediated trehalose transport into hemolymph, thereby maintaining trehalose homeostasis. These findings collectively demonstrate that BmERR plays a potential regulatory role in maintaining trehalose metabolic.

Trehalose metabolism is a pathway unique to insects, and its regulation by ERR in silkworms underscores the broader versatility of ERR functions compared to vertebrates. The study has several limitations. First, the identification of ERR-binding elements relied on computational promoter analysis, which, while informative, requires experimental validation to confirm functional interactions. Second, the study primarily focused on a subset of metabolic pathways, leaving the broader regulatory network of ERR in silkworms unexplored. Third, the findings are based on a single model organism; Future studies should investigate whether these regulatory mechanisms are conserved across other lepidopterans or insect species.

This study used silkworms as a model organism to preliminarily outline the ERR metabolic regulatory network, highlighting the potential role of ERR in insect energy metabolism. The findings lay the groundwork for future research on the metabolic regulatory functions of ERR in insects. Given the differences in energy metabolism between insects and vertebrates, as well as the complexity of ERR’s functional network, further studies are needed. Future studies should integrate advanced approaches such as gene editing, overexpression, and omics technologies to validate and characterize ERR target genes in lipid, amino acid, and carbohydrate metabolism pathways.

## 5. Conclusions

The gene promoters involved in lipid, amino acid, and carbohydrate metabolism pathways in silkworms contain elements that can bind to ERR. Notably, BmERR binds to the ERRE on the promoter of the *BmTret1* to regulate trehalose homeostasis, underscoring its critical role in energy metabolism. These findings emphasize ERR’s broader and more significant role in insects, contributing to a deeper understanding of insect metabolism and its potential applications.

## Figures and Tables

**Table 1:** Functions of ERR in insects.

The Founds and Funtions of ERR in Insects		
Order	Species	Funtions
*Lepidoptera*	*Bombyx mori*	Nutrition [6] Glycolysis [24] Trehalose metabolism [30] 20E affects ERR expression [31]
	*Agrotis ipsilon*	Sexual behavior [28]
*Diptera*	*Drosophila melanogaster*	Glycolysis, pentose phosphate pathway, lipid metabolism, and carbohydrate metabolism [21,22,32] ERR is involved in hypoxia response of HIF [23] Ethyl p-hydroxybenzoate affects ERR expression [33] Growth and development of sexual organs [26,34] BPA affects ERR expression
	*Aedes aegypti*	Ecdysone Carbohydrate metabolism, lipid metabolism and glucose metabolism [35]
	*Chironomus riparius*	BPA, NP and DEHP affects ERR expression [36] TCS affects ERR expression [37]
*Hymenoptera*	*Polyrhachis vicina Roger*	Growth and development of sexual organs, gut morphology, reproductive processes, and digestive activities [38] Nutrition and juvenile hormone [39]
	*Apis cerana cerana*	Abiotic stress responses [8]
*Hemiptera*	*Nilaparvata lugens*	20E and JH [30]
*Orthoptera*	*Teleogryllus emma*	Growth and development of nerves and the morphology of male reproductive organs [7]
*Homoptera*	*Myzus persicae*	Glycolysis [27] 3-Methylcatechol affects ERR expression [40]

**Table 2:** Metabolic gene annotation and binding sites that can bind to BmERR DBD protein.

Metabolic Type	Gene_ID	Gene Name	Gene Description	Binding Motifs
Trehalose metabolism	KWMTBOMO07250	GlyP	glycogen phosphorylase	CAACGTCA
	KWMTBOMO07450	Tps	trehalose-6-phosphate synthase	CAAGGTTA
	KWMTBOMO16046	Tret1	trehalose transporter	CTCACAAGGTCC
Lipid metabolism	KWMTBOMO08103	Atgl	adipose triglyceride lipase	CAATGTCA
	KWMTBOMO00223	GK	glycerol kinase	GAAGGTCA CAATGTCAATGTC
	KWMTBOMO03651	Acyl	acyl-CoA	TGCCCTTG
Amino acid metabolism	KWMTBOMO01118	FAH	fumarylacetoacetate hydrolase	CCAAGGACGT
	KWMTBOMO04345	HPD	4-hydroxyphenylpyruvate dioxygenase	AAAGGGCG ACGAGGTTAT AAACGTCA
	KWMTBOMO12353	Chsy1	chondroitin sulfate synthase 1	CAAGGTTG
Carbohydrate metabolism	KWMTBOMO00773	G6pase	glucose-6-phosphatase	AAATGTCA
	KWMTBOMO04913	Ca7	carbonic anhydrase 7	CAAAGTCA CAAGGGCA CGAATGTCAT ATAGGTTA
	KWMTBOMO07147	Aldh1b1	aldehyde dehydrogenase 1 B1	CTAACCTTAA
	KWMTBOMO00939	akr2e	aldo-keto reductase family 2 member E4	TTAAGGTTAC
	KWMTBOMO16421	UGT33D8	UDP-glycosyltransferase UGT33D8	CAAGGTCA
	KWMTBOMO07079	UGT46A2	UDP-glycosyltransferase UGT46A2	TTAAGGTTAC

## Data Availability

The original contributions presented in the study are included in the article/Supplementary Materials; further inquiries can be directed to the corresponding author.

## Supplementary Materials

The following supporting information can be downloaded at: https://www.mdpi.com/article/10.3390/insects16050469/s1, Table S1: Number of ERRE-like elements with a score greater than 85 on the promoters of metabolism-related genes. Table S2: Probe sequences used in Electrophoretic Mobility Shift Assay (EMSA) for metabolism-related genes in the silkworm.
