# Supplementary material for: Estrogen-Related Receptor Potential Target Genes in Silkworm (Bombyx mori): Insights into Metabolic Regulation
Source: Insects. 2025 Apr 29;16(5):469. doi: 10.3390/insects16050469 (PMC12112554; doi:10.3390/insects16050469)
Supplement: Supplementary file 1 [file insects-16-00469-s001.zip › insects-3514303-supplementary.pdf]

Table S1 Number of ERRE-like elements with a score greater than 80 on the promoters of metabolism-related genes

| | Gene_ID | Gene name | Gene description | Number of ERRE |
|---|---|---|---|---|
| Glycolysis | KWMTBOMO13777 | HK | Hexokinase | 7 |
| | KWMTBOMO02749 | Pfk | ATP-dependent 6-phosphofructokinase isoform X1 | 8 |
| | KWMTBOMO09555 | Ald | fructose-bisphosphate aldolase isoform X1 | 7 |
| | KWMTBOMO09553 | Fba | Fructose 1,6-bisphosphate aldolase | 7 |
| | KWMTBOMO00254 | Tpi | Triosephosphate isomerase | 5 |
| | KWMTBOMO01182 | Gapdh | Glyceraldehyde-3-phosphate dehydrogenase | 7 |
| | KWMTBOMO08760 | Pgk | Phosphoglycerate kinase | 13 |
| | KWMTBOMO09934 | Pgm | Phosphoglyceromutase | 9 |
| | KWMTBOMO04605 | Eno1 | Enolase1 | 4 |
| | KWMTBOMO00493 | Eno2 | Enolase2 | 13 |
| | KWMTBOMO05295 | Pyk | Pyruvate kinase isoform X1 | 8 |
| Tricarboxylic acid cycle | KWMTBOMO08241 | Pdh | Pyruvate dehydrogenase | 7 |
| | KWMTBOMO00279 | CS2 | Citrate synthase 2 | 7 |
| | KWMTBOMO10508 | Aco | Aconitate hydratase | 6 |
| | KWMTBOMO05820 | Idh | Isocitrate dehydrogenase | 9 |
| | KWMTBOMO09054 | Idhp | Isocitrate dehydrogenase precursor | 8 |
| | KWMTBOMO11127 | FC | Fumarase C C-terminus | 3 |
| | KWMTBOMO01417 | MDH | Malate dehydrogenase | 7 |
| Trehalose metabolism | KWMTBOMO10093 | Treh1p | Trehalase1 precursor | 8 |
| | KWMTBOMO10092 | Treh1 | Trehalase1 | 6 |
| | KWMTBOMO16497 | Treh2p | Trehalase-2 precursor | 7 |
| | KWMTBOMO16054 | Treh2 | Trehalase-2_isoform_X1 | 9 |
| | KWMTBOMO16046 | Tret1 | Trehalose transporter1 | 10 |
| | KWMTBOMO07450 | TPS | Trehalose-6-phosphate synthase | 7 |
| | KWMTBOMO07250 | Glyp | Glycogen phosphorylase | 10 |
| Carbohydrate metabolism | KWMTBOMO00773 | G6pase | Glucose-6-phosphatase | 6 |
| | KWMTBOMO14536 | ACLY | ATP-citrate lyase | 8 |
| | KWMTBOMO00939 | akr2e | Aldo-keto reductase AKR2E4-like | 8 |
| | KWMTBOMO09561 | akr2e4X1 | Aldo-keto reductase AKR2E4 isoform X1 | 9 |
| | KWMTBOMO06066 | UGT48C1 | UDP-glycosyltransferase UGT48C1 precursor | 5 |

| | | | | |
|---|---|---|---|---|
| | KWMTBOMO12649 | Sut | Sucrase-isomaltase, intestinal-like | 9 |
| | KWMTBOMO07079 | UGT46A2 | UDP-glycosyltransferase UGT46A2 precursor | 6 |
| | KWMTBOMO00984 | akr | Aldo-keto reductase | 7 |
| | KWMTBOMO13037 | CRYL | Lambda-crystallin isoform X1 | 8 |
| | KWMTBOMO07147 | Aldh1b1 | Aldehyde dehydrogenase 1 B1 | 10 |
| | KWMTBOMO10066 | Chit10 | Chitinase 10 | 7 |
| | KWMTBOMO02040 | chs-2 | Chitin synthase | 7 |
| | KWMTBOMO02041 | Chit | Chitin synthase A isoform X3 | 8 |
| | KWMTBOMO16421 | UGT33D8 | UDP-glycosyltransferase UGT33D8 | 9 |
| | KWMTBOMO04913 | Ca7 | Carbonic anhydrase 7 | 8 |
| | KWMTBOMO12764 | pik3c3 | Phosphatidylinositol 3-kinase catalytic subunit type 3 | 6 |
| Lipid metabolism | KWMTBOMO08103 | Atgl | Adipose triglyceride lipase | 10 |
| | KWMTBOMO06932 | Fasn2 | Fatty acid synthase 2 | 8 |
| | KWMTBOMO00223 | GK | Glycerol kinase | 7 |
| | KWMTBOMO14074 | Acc | Acetyl-CoA carboxylase | 3 |
| | KWMTBOMO10006 | Lal | Lysosomal acid lipase-1 | 2 |
| | KWMTBOMO03651 | Acyl | Acyl-CoA desaturase-like | 9 |
| | KWMTBOMO15287 | Elovl | Elongation of very-long-chain fatty acid protein | 7 |
| | KWMTBOMO04287 | Lip1 | Lipase 1 | 5 |
| | KWMTBOMO09851 | Fas | Fatty acid synthase | 9 |
| | KWMTBOMO09857 | TE-domain | Thioesterase domain | 10 |
| | KWMTBOMO11288 | Akhr | Adipokinetic hormone receptor | 4 |
| | KWMTBOMO11286 | Akhr1 | Adipokinetic hormone receptor isoform X1 | 6 |
| | KWMTBOMO07031 | FarX1 | Fatty-acyl reductase isoform X1 | 7 |
| | KWMTBOMO14199 | Far | Fatty acyl-CoA reductase wat-like | 7 |
| Amino acid metabolism | KWMTBOMO07599 | GNMT | Glycine N-methyltransferase | 5 |
| | KWMTBOMO12353 | Chsy1 | Chondroitin sulfate synthase1 | 5 |
| | KWMTBOMO16447 | Hykk | Hydroxylysine kinase | 10 |
| | KWMTBOMO04345 | HPD | 4-Hydroxyphenylpyruvate dioxygenase | 5 |
| | KWMTBOMO04438 | DNMT1 | D cytosine-5 methyltransferase isoform X1 | 5 |
| | KWMTBOMO01118 | FAH | Fumarylacetoacetate hydrolase | 6 |
| Detoxification | KWMTBOMO11596 | Hsp1 | Heat shock protein 1 | 7 |
| | KWMTBOMO09944 | CYP6AE7 | Cytochrome P450 CYP6AE7 | 10 |
| | KWMTBOMO09952 | CYP6AE2 | Cytochrome P450 CYP6AE2 | 10 |
| | KWMTBOMO09951 | CYP6B5 | Cytochrome P450 CYP6B5 | 10 |

| | | | |
|---|---|---|---|
| KWMTBOMO07943 | CYP4G15 | Cytochrome P450 CYP4G15 | 10 |
| KWMTBOMO10808 | AOX1 | Aldehyde oxidase 1 | 8 |
| KWMTBOMO13530 | Sod | Superoxide dismutase | 9 |
| KWMTBOMO16177 | PHYH | Phytanoyl-CoA dioxygenase | 9 |

Table S2 Probe sequences used in Electrophoretic Mobility Shift Assay (EMSA) for metabolism-related genes in the silkworm.

| **Gene name** | **Name** | **Site** | **Sequence (5'-3')** |
|---|---|---|---|
| GK | ERRE-like CRE1 Probe | -1950~-1957(-) | F: CCCCATGACCTTCGCCGG<br>R: CCGGCGAAGGTCATGGGG |
| | ERRE-like CRE2 Probe | -815~-822(-) | F: GTCTATGAGCTTGGGTGA<br>R: TCACCCAAGCTCATAGAC |
| | ERRE-like CRE3 Probe | 185~-172(+) | F: GATTGTCAATGTCAATGTCATGAACA<br>R: TGTTCATGACATTGACATTGACAATC |
| Acyl | ERRE-like CRE1 Probe | -1927~-1934(-) | F: TATATTGCCCTTGTAAGT<br>R: ACTTACAAGGGCAATATA |
| | ERRE-like CRE2 Probe | -1798~-1789(+) | F: TTTGAAGAAGGACATGTCAT<br>R: ATGACATGTCCTTCTTCAAA |
| | ERRE-like CRE3 Probe | -903~-896(+) | F: GCGTTAATGGTCATGGTA<br>R: TACCATGACCATTAACGC |
| Atgl | ERRE-like CRE1 Probe | -1679~-1686(-) | F: GTACCTACCCTTTATCTT<br>R: AAGATAAAGGGTAGGTAC |
| | ERRE-like CRE2 Probe | -1174~1183(-) | F: CTCATACGCCCTTCAGGTCA<br>R: TGACCTGAAGGGCGTATGAG |
| | ERRE-like CRE3 Probe | -67~-74(-) | F: CGCTCTGACATTGAAAAG<br>R: CTTTTCAATGTCAGAGCG |
| FAH | ERRE-like CRE1 Probe | -429~-420(+) | F: TTTTTCCAAGGACGTTTGAG<br>R: CTCAAACGTCCTTGGAAAAA |
| | ERRE-like CRE2 Probe | -217~-224(-) | F: AATAGTGACCTATGGATA<br>R: TATCCATAGGTCACTATT |
| | ERRE-like CRE3 Probe | -176~-169(+) | F: GTTTGAAAGTTCATCCTT<br>R: AAGGATGAACTTTCAAAC |
| HPD | ERRE-like CRE1 Probe | -1504~-1511(-) | F: ACACGCGCCCTTT ATATA<br>R: TATATAAAGGGCGCGTGT |
| | ERRE-like CRE2 Probe | -1041~-1050(-) | F: GGACTATAACCTCGT CTCTT<br>R: AAGAGACGAGGTTATAGTCC |
| | ERRE-like CRE3 Probe | -168~-161(+) | F: TCCACAAACGTCA ATAGC<br>R: GCTATTGACGTTTGTGGA |
| Chsy1 | ERRE-like CRE1 Probe | -1774~-1767(+) | F: TCTCACAAGGTTGATGTC<br>R: GACATCAACCTTGTGAGA |
| | ERRE-like CRE2 Probe | -508~-501(+) | F: TGGGACAAGTTCACATCG<br>R: CGATGTGAACTTGTCCCA |
| Aldh1b1 | ERRE-like | -1694~-1687(+) | F: GATTAAAAGGTAAATACG |

| | | | |
|---|---|---|---|
| | CRE1 Probe | | R: CGTATTTACCTTTTAATC |
| | ERRE-like CRE2 Probe | -1249~-1256(-) | F: ATTGATAACCTTTATTGC<br>R: GCAATAAAGGTTATCAAT |
| | ERRE-like CRE3 Probe | -357~-348(+) | F: CCTGGTTAAGGTTAGAAGTC<br>R: GACTTCTAACCTTAACCAGG |
| Akr2e | ERRE-like CRE1 Probe | -1663~-1670(-) | F: TTTCTCAACCTTTTTTGT<br>R: ACAAAAAAGGTTGAGAAA |
| | ERRE-like CRE2 Probe | -712~-703(+) | F: ACTAGTTAAGGTTACGCTGA<br>R: TCAGCGTAACCTTAACTAGT |
| | ERRE-like CRE3 Probe | -25~-18(+) | F: AAATCCAAGATCATCTAT<br>R: ATAGATGATCTTGGATTT |
| G6pase | ERRE-like CRE1 Probe | -1943~-1936(+) | F: GCTTGAAAGGTAAAAGTG<br>R: CACTTTTACCTTTCAAGC |
| | ERRE-like CRE2 Probe | -802~-809(-) | F: TTGTTTGACATTTAGGTC<br>R: GACCTAAATGTCAAACAA |
| | ERRE-like CRE3 Probe | -27~-34(-) | F: AAGTATGACATTTTGACATTTGACAT<br>R: ATGTCAAATGTCAAAATGTCATACTT |
| Ca7 | ERRE-like CRE1 Probe | -1490~-1497(-) | F: TAATGTGACTTTGAATCG<br>R: CGATTCAAAGTCACATTA |
| | ERRE-like CRE2 Probe | -1066~-1073(-) | F: TTTATTGCCCTTGTAGGC<br>R: GCCTACAAGGGCAATAAA |
| | ERRE-like CRE3 Probe | -899~-890(+) | F: GACTTCGAATGTCATGTCGC<br>R: GCGACATGACATTCGAAGTC |
| | ERRE-like CRE4 Probe | -68~-31(+) | F: ATTCAATAGGTTATTTCT<br>R: AGAAATAACCTATTGAAT |
| UGT46A2 | ERRE-like CRE1 Probe | -1182~-1189(-) | F: TTGTTTGATCTTTGGTTT-<br>R: AAACCAAAGATCAAACAA |
| | ERRE-like CRE2 Probe | -780~-787(-) | F: TCGTCGTAACCTTAA AGATA<br>R: TATCTTTAAGGTTACGACGA |
| UGT33D8 | ERRE-like CRE1 Probe | -1756~-1749(+) | F: CGACTTAAGGTCACATTT<br>R: AAATGTGACCTTAAGTCG |
| | ERRE-like CRE2 Probe | -78~-71(+) | F: TCAGGCAAGGTCAAAGCC<br>R: GGCTTTGACCTTGCCTGA |
| TPS | ERRE-like CRE1 Probe | -1369~-1376(-) | F: TTGCTTGACCTTATTGTT<br>R: AACAATAAGGTCAAGCAA |
| | ERRE-like CRE2 Probe | -785~-778(+) | F: ACAAACAAGTTCAAACAA<br>R: TTGTTTGAACTTGTTTGT |
| | ERRE-like CRE3 Probe | -250~-257(-) | F: GGCTGTAACCTTGTCCTC<br>R: GAGGACAAGGTTACAGCC |
| Glyp | ERRE-like CRE1 Probe | -1146~-1153(-) | F: TCTAGTGACCGTTGACCG<br>R: CGGTCAACGGTCACTAGA |
| | ERRE-like CRE2 Probe | -645~-652(-) | F: TTTTATGACCTGGTAACT<br>R: AGTTACCAGGTCATAAAA |

| | | | |
|---|---|---|---|
| | ERRE-like CRE3 Probe | -157~-150(+) | F: ACGTCCAACGTCAAACCA<br>R: TGGTTTGACGTTGGACGT |
| Tret1 | ERRE-like CRE1 Probe | -1396~-1408(-) | F: AGCTTCTCCTTGACGTGTAG<br>R: CTACACGTCAAGGAGAAGCT |
| | ERRE-like CRE2 Probe | -825~-812(-) | F: TTTTTGTAACCTTAAACTCCT<br>R: AGGAGTTTAAGGTTACAAAAA |
| | ERRE-like CRE3 Probe | -416~-404(+) | F: GCGGACTCACAAGGTCCTACCA<br>R: TGGTAGGACCTTGTGAGTCCGC |

(-): antisense strand; (+): sense strand.
